# Changes in self-reported risky sexual behaviour indicators among adults receiving regular risk reduction counselling and optional initiation of pre-exposure prophylaxis in an HIV vaccine preparedness study in Masaka, Uganda

**DOI:** 10.1080/16549716.2023.2242672

**Published:** 2023-08-07

**Authors:** Jonathan Kitonsa, Sheila Kansiime, Sylvia Kusemererwa, Martin Onyango, Berna Nayiga, Anita Kabarambi, Joseph O Mugisha, Pontiano Kaleebu, Eugene Ruzagira

**Affiliations:** a Medical Research Council/Uganda Virus Research Institute and London School of Hygiene and Tropical Medicine, Uganda Research Unit, Entebbe, Uganda; b Research, International Centre for Child Health and Development (ICHAD), Masaka, Uganda; c Infection Biology, London School of Hygiene and Tropical Medicine, London, UK; d Epidemiology, London School of Hygiene and Tropical Medicine, London, UK

**Keywords:** Risky sexual behaviour, HIV, pre-exposure prophylaxis, risk reduction counselling

## Abstract

**Background:**

HIV risk reduction counselling may reduce risk-taking behaviours. Yet, concerns remain about risk compensation among individuals initiating pre-exposure prophylaxis (PrEP).

**Objective:**

We assessed changes in risky sexual behaviour indicators among HIV vaccine preparedness study participants who received regular risk reduction counselling and referral for PrEP in Masaka, Uganda.

**Methods:**

Adults (18–39 years) at high risk of HIV infection were enrolled in the study between July 2018 and December 2021. Data were collected on socio-demographic factors (baseline) and self-reported sexual risk behaviours (baseline, six monthly). HIV testing and risk-reduction counselling and referral for PrEP were done quarterly. Participants who had completed at least 1 year of follow-up were included in the analysis. Proportional differences and McNemar chi-square tests were used to assess changes in the prevalence of self-reported risky sexual behaviour indicators between baseline and 1 year. Logistic regression was used to assess the predictors of unchanged/increased HIV risk at 1 year.

**Results:**

Three hundred participants [132 (44%) females, 152 (51%) aged ≤24 years] were included in this analysis. Eighty-one (27%) participants initiated PrEP at 1 year. Compared to baseline, there were significant reductions in the prevalence of the following self-reported HIV risk indicators at 1 year (overall, among non-PrEP initiators, and among PrEP initiators): transactional sex, ≥6 sexual partners, unprotected sex with ≥3 partners, sex while drunk, and sexually transmitted infection diagnosis/treatment. Percentage differences ranged from 10% for individuals reporting at least six sexual partners to 30% for those reporting unprotected sex with three or fewer sexual partners. There was weak evidence of association between female gender and unchanged/increased HIV risk at 1 year (adjusted OR: 1.35, 95% CI (0.84–2.17)). No other indicators, including PrEP use, were associated with unchanged/increased HIV risk at 1 year.

**Conclusion:**

Regular risk-reduction counselling may reduce risky sexual behaviour, while PrEP initiation may not lead to risk compensation.

## Introduction

The global incidence of HIV remains high despite the increasing number of proven HIV prevention tools. Incidence is highest among key populations such as men who have sex with men, commercial sex workers, long-distance truck drivers, and fisher-folk communities [1]. In 2020, individuals from key populations and their sexual partners accounted for approximately 65% and 39% of all new HIV infections globally and in Southern and Eastern Africa, respectively [1]. The perpetuation of the HIV epidemic through key populations has remained a critical issue especially in sub-Saharan Africa, where the annual incidence of HIV among key populations is reported to be higher than 20/100 person years in some settings [2]. In Uganda, HIV prevalence among female commercial sex workers has been previously estimated to be 31.3%, higher than the national average of 5.4 [3].

An effective response to the HIV epidemic requires the recruitment of populations at high risk of HIV into HIV prevention programmes, where they can receive information and services tailored to them [4]. One of the measures that could promote HIV risk reduction in at-risk populations is a well-packaged counselling programme. However, evidence on the impact of counselling on risky sexual behaviour remains inconclusive [5–7]. In addition, there are concerns about the use of pre-exposure prophylaxis (PrEP), a key HIV prevention tool, causing behavioural risk compensation, i.e. increased risk-taking behaviours due to a decrease in perceived risk [8–10]. While the attribution of increased risky behaviour to the use of prevention methods for HIV remains unconfirmed, global trends showing increased incidence of sexually transmitted infections (STIs) in the last few years make this proposition worth investigating [11,12]. In the current study, we assessed changes in self-reported risky sexual behaviour indicators in an HIV vaccine preparedness study in which participants were provided regular risk reduction counselling and optional referrals and initiation of PrEP.

## Methods

### Study design and setting

Data used for this analysis are from the PrEPVacc registration cohort – a prospective longitudinal study intended to identify, recruit, follow-up, and prepare HIV-negative individuals at high risk of HIV infection for participation in the PrEPVacc HIV vaccine efficacy trial (NCT04066881) [13,14]. The registration cohort was initiated in July 2018 and is being conducted at five clinical research sites in four African countries: Durban, South Africa; Mbeya and Dar es Salaam, Tanzania; Maputo, Mozambique; and Masaka, Uganda. This analysis included participants enrolled at the Masaka site. The site is in Masaka city, Masaka district, approximately 120 km south-west of Kampala, Uganda’s capital. Masaka district has multiple ‘hot spots’ for high-risk sexual activity located in fishing communities along Lake Victoria and several trading centres along the Trans-African Highway connecting Kenya to the Democratic Republic of Congo and Rwanda through Uganda. HIV prevalence in Masaka and the neighbouring districts is 11.7%, one of the highest in the country [3].

### Study participants

Individuals were eligible to participate in the PrEPVacc registration cohort study if they were aged 18–39 years, HIV-uninfected, able and willing to provide informed consent, willing to provide locator information and be available for follow-up, willing to undergo regular HIV testing, and at high risk of HIV infection. High risk for HIV infection was defined as having any one of the following: suspected/confirmed STI, unprotected sex (sex without a condom) with ≥2 partners, unprotected sex with a new partner in the past 3 months, or unprotected sex in exchange for money/goods in the past month. The questionnaire that was used for risk assessment is provided as supplementary information S1. Individuals who were participating in other HIV prevention studies were excluded. This analysis only included participants that had completed 1 year of follow-up by December 2021.

### Study procedures

Potential study participants were identified through community-based HIV counselling and testing services. Individuals who tested HIV-negative were invited to the study clinic for eligibility screening. Screening procedures consisted of provision of detailed study information, obtaining written informed consent, HIV testing, and risk reduction counselling according to the national HIV counselling guidelines [15] and the site Standard Operating Procedures for HIV counselling, eligibility assessment, and enrolment for those determined to be eligible. Enrolled participants completed an interviewer-administered questionnaire on social demographics, HIV risk, and reduction behaviours including awareness of and use of oral PrEP. Participants were also given information and counselling on oral PrEP and those willing to initiate oral PrEP, referred to a PrEP provider of their choice. Except for transport costs to reach the providers, PrEP was offered at no cost to the participant at all provider facilities. Participants were referred to facilities offering PrEP that were nearest and favourable to them.

During follow-up, participants underwent HIV testing every 3 months and completed interviewer-administered questionnaires on HIV risk and reduction behaviours every 6 months. Risk reduction counselling, provision of information on and referrals for PrEP, where necessary, were performed at all follow-up visits. If a participant was assessed and found to no longer be at high risk of HIV acquisition, they were discontinued from follow-up, since they then did not qualify to be enrolled in the PrEPVacc HIV vaccine efficacy trial.

### Procedure for risk reduction counselling

The risk assessment questionnaire administered by a counsellor enabled determination of an individual’s level of risk. Risk triggers for the identified risk factors for the particular participant were then determined, e.g. excessive alcohol consumption and/or illicit drug use affecting decision-making, leading to unprotected sex. The counsellor then asked the client to identify things he or she could reasonably do to reduce on the identified risk(s). If the client was unable to make suggestions, the counsellor could make recommendations. The client and counsellor then identified potential barriers to the implementation of these suggestions. The two then went ahead to identify sources of support for the client to promote behavioural change, i.e. a person that could support the client to change behaviour. The counsellor then built confidence in the client on his or her ability to carry out this behavioural change plan. All this was then documented in the participant records and revisited on subsequent visits.

### Sample size

Assuming an underlying baseline prevalence of 50% for a given risky sexual behaviour indicator, we estimated that 300 participants would provide >80% power to detect a change of ≥15% between the baseline and 1-year prevalence of the indicator. A baseline prevalence lower or higher than 50% indicator would provide even more power to detect a similar change in its prevalence between the two time points.

### Statistical analysis

Data were managed in OpenClinica (Community Edition) and exported to STATA (College Station, TX, version 15.0) for analysis. Participants’ characteristics were summarised using frequencies and percentages for categorical variables and means (standard deviations) and/or medians (interquartile ranges) for continuous variables. Changes in HIV risk indicators between baseline and 1 year were determined using proportion differences and McNemar chi-square tests. To determine predictors of change in HIV risk between baseline and 1 year, participants were assigned a summative risk score, where each risk indicator was assigned a score of 1 for risk and 0 if no risk [condom use at last sex (No/Not sure = 1, Yes = 0); transactional sex (No = 0, Yes = 1); ≥6 sexual partners (No = 0, Yes = 1); unprotected sex with ≥3 partners (No = 0, Yes = 1); sex while drank (No = 0, Yes = 1); used recreational drugs (No = 0, Yes = 1); diagnosed/treated for STI (No/Don’t know = 0, Yes = 1)].

A change variable categorised as decrease in risk [0], and unchanged or increased risk [1] was created and used to estimate the difference between a participant’s risk score at baseline vs 1 year. Unchanged and increased risks were categorised together, because they were both unfavourable outcomes. Univariable and multivariable logistic regression models were used to determine predictors of unchanged/increased HIV risk between baseline and 1 year. Variables with a p-value of <0.2 at bivariate analysis were included in the multivariable model.

### Ethics and consent

The PrEPVacc registration cohort study protocol was approved by the Uganda Virus Research Institute Research Ethics Committee (GC/127/18/03/637), the Uganda National Council for Science and Technology (HS2339), and London School of Hygiene & Tropical Medicine Ethics Committee (26494-1). Written informed consent was obtained from participants before any study procedures were conducted. Individuals who tested HIV-positive at any of the study visits were provided post-test counselling and referred for HIV care. Additionally, pregnant HIV-positive female participants were referred to prevention-of-mother-to-child HIV transmission services. Individuals who had STI symptoms and/or signs were treated using the Uganda national guidelines on syndromic management of STIs.

## Results

### Baseline characteristics and PrEP uptake

**Figure 1.**
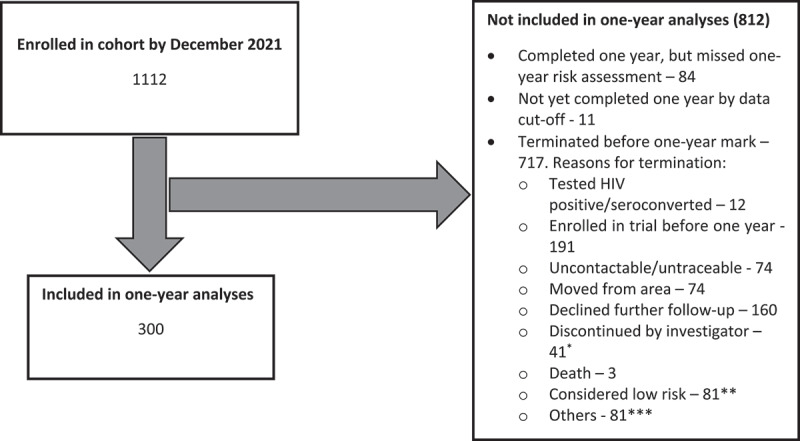
Flow diagram showing participants included/excluded from analyses.

As of December 2021, a total of 1112 individuals had been enrolled in the PrEPVacc registration cohort; of whom, 300 were included in this analysis (Figure 1). Some participants were not included in this analysis, because they missed the 1-year risk assessment (*n* = 84), had not yet completed 1 year by data cut-off (*n* = 11), were terminated before the 1-year visit (*n* = 717).

*The investigator had the option to discontinue a participant, for example, if they were not adhering to protocol requirements, deemed to be offering false information, etc.

**81 participants enrolled in the cohort were excluded from this analysis, because they did not complete 1 year of follow-up, as their risk status changed to low risk. Their risk had been high at enrolment.

***For example, those that turned 39 years prior to enrolment into the clinical trial, became pregnant, were unwilling to continue using contraception, etc.

A comparison of participants with 1-year follow-up and those without has been shared as supplementary information S2.

Among those included in the analysis, the mean (SD) age was 26 (6.13) years, 132 (44%) were females, 152 (51%) were aged ≤24 years, 195 (65%) had primary school education or less, and 39 (13%) reported sex work as their main occupation (Table 1). Eighty-one (27%) participants had initiated PrEP by 1 year.

**Table 1.** Participants’ baseline social demographic characteristics and PrEP uptake after 1 year of follow-up.

Socio-demographic characteristics	All participants	Initiated PrEP
	N (%)^†^	N (%)^†^
All	300 (100)	81 (100)
Gender		
Male	168 (56)	42 (52)
Female	132 (44)	39 (48)
Age category		
≤24	152 (51)	35 (43)
25–34	114 (38)	32 (40)
≥35	34 (11)	14 (17)
Education		
≤Primary	195 (65)	54 (67)
≥Secondary	105 (35)	27 (33)
Marital status		
Single	142 (48)	39 (48)
Married/cohabiting/in a relationship	121 (40)	33 (41)
Divorced/separated/widowed	37 (12)	9 (11)
Religion		
Christian	229 (76)	59 (73)
Muslim/other	71 (24)	22 (27)
Occupation*		
Sex work	39 (13)	18 (22)
Subsistence fisheries	64 (21)	18 (22)
Salon/lodge/bar worker, market/street vendor	82 (27)	22 (27)
Other occupation (professional, student, crafts etc.)	130 (43)	29 (36)
Electricity in household		
No	82 (27)	24 (30)
Yes	218 (73)	57 (70)

^†^Column percentage; *Multiple options allowed.

### Changes in risky sexual behaviour indicators between baseline and 1 year

All risky sexual behaviours at baseline were more prevalent among those that subsequently took up PrEP. Compared to baseline, there were reductions in the prevalence of the following self-reported risky sexual behaviour indicators at 1 year: transactional sex [overall (68%–50%, *P* < 0.001), non-PrEP initiators (64%–45%, *P* < 0.001), PrEP initiators (80%–64%, *P* = 0.016)]; ≥6 sexual partners [overall (33%–23%, *P* < 0.001), non-PrEP initiators (27%–17%, *P* = 0.002), PrEP initiators (48%–38%, *P* = 0.059)]; unprotected sex with ≥3 partners [overall (52%–24%, *P* < 0.001), non-PrEP initiators (50%–20%, *P* < 0.001), PrEP initiators (59%–35%, *P* < 0.001)]; sex while drunk [overall (45%–31%, *P* < 0.001), non-PrEP initiators (42%–31%, *P* = 0.003), PrEP initiators (54%–33%, *P* = 0.002)]; diagnosed/treated for STI [overall (39%–17%, *P* < 0.001), non-PrEP initiators (37%–15%, *P* < 0.001), PrEP initiators (47%–21%, *P* < 0.001)]. No differences were observed in the prevalence of condom use at last sex and use of recreational drugs between baseline and 1 year in all groups (Table 2). Changes in risk scores are provided as supplementary information (S3).

**Table 2.** Change in prevalence of HIV risk behaviour indicators at 1 year, overall and by PrEP uptake status.

Self-reported HIV risk indicator (last 3 months)	All (*N* = 300)					Did not initiate PrEP (*N* = 219)					Initiated PrEP (*N* = 81)				
	Baseline, n (%)	1 year, n (%)	% Diff (95% CI)	*McNemar’s* *chi-square*	*P*-value	Baseline, n (%)	1 year, n (%)	% Diff (95% CI)	*McNemar’s* *chi-square*	*P*-value	Baseline, n (%)	1 year, n (%)	% Diff (95% CI)	*McNemar’s* *chi-square*	*P*-value
Condom use at last sex	67 (22)	75 (25)	3 (−4, 9)	0.78	0.377	46 (21)	51 (23)	2 (−5, 9)	0.5	0.500	21 (26)	24 (30)	4 (−10, 17)	0.3	0.563
Transactional sex	205 (68)	151 (50)	−18 (−25, −11)	25.1	<0.001	140 (64)	99 (45)	−19 (−27, −10)	19.3	<0.001	65 (80)	52 (64)	−16 (−30, −2)	5.8	0.016
≥6 sexual partners	98 (33)	66 (23)	−10 (−16, −4)	12.9	<0.001	59 (27)	36 (17)	−10 (−16, −3)	9.4	0.002	39 (48)	30 (38)	−10 (−22, 1)	3.6	0.059
Unprotected sex with ≥3 partners	157 (52)	70 (24)	−28 (−35, −22)	62.3	<0.001	109 (50)	43 (20)	−30 (−37, −22)	48.1	<0.001	48 (59)	27 (35)	−24 (−39, −12)	14.3	<0.001
Sex while drunk	136 (45)	94 (31)	−14 (−21, −7)	17.0	<0.001	92 (42)	67 (31)	−11 (−19, −3)	8.6	0.003	44 (54)	27 (33)	−21 (−35, −7)	9.3	0.002
Used recreational drugs	38 (13)	33 (11)	−2 (−5, 21)	0.93	0.336	24 (11)	21 (10)	−1 (−5, 3)	0.53	0.467	14 (17)	12 (15)	−2 (−11, 6)	0.4	0.527
Diagnosed/treated for STI	118 (39)	50 (17)	−22 (−29, −16)	40.6	<0.001	80 (37)	33 (15)	−22 (−29, −14)	28.7	<0.001	38 (47)	17 (21)	−26 (−41, −11)	11.9	<0.001

n, number; STI, sexually transmitted infection; % Diff, percentage difference.

Median risk score (IQR) overall at baseline and 1 year was 3 (2,3,4) and 2 (1,2,3), respectively. Median risk score (IQR) at baseline and 1 year respectively was 3 (2,3,4) and 2 (1,2,3) among those who did not initiate PrEP and 4 (3,4,5) and 2 (2,3,4) among those who initiated PrEP.

### Predictors of unchanged/increased HIV risk

One hundred and fifteen (38%) participants had an unchanged/increased overall HIV risk score between baseline and 1 year (Table 3). There was a higher proportion of participants with an unchanged/increased risk score among those not on PrEP than those on PrEP (40% vs 35%, P-value = 0.415). Despite weak evidence of association, women were more likely to have unchanged/increased risk than men at 1 year (adjusted OR: 1.35, 95% CI (0.84–2.17)).

**Table 3.** Predictors of unchanged/increased HIV risk at 1 year.

Socio-demographic characteristics	Number (%)	Number (%) with no change/increase in risk score^#^	Univariable analysis		Multivariable analysis	
			OR (95% CI)	P-value	OR (95% CI)	P-value
Overall	300 (100)	115 (38)				
Gender						
Male	168 (56)	59 (35)	Ref		Ref	
Female	132 (44)	56 (42)	1.36 (0.85–2.17)	0.197	1.35 (0.84–2.17)	0.212
Age (years) (mean, SD)	(26, 6.13)	(26, 6.39)	0.99 (0.95–1.03)	0.684	1.00 (0.96–1.03)	0.806
Education						
≤Primary	195 (65)	71 (36)	Ref			
≥Secondary	105 (35)	44 (42)	1.26 (0.78–2.05)	0.351		
Marital status						
Single	142 (47)	52 (37)	Ref			
Married/cohabiting/in a relationship	121 (40)	49 (40)	1.18 (0.72–1.94)			
Divorced/separated/widowed	37 (12)	14 (38)	1.05 (0.50–2.22)	0.811		
Religion						
Christian	229 (76)	88 (38)	Ref			
Muslim/other	71 (24)	27 (38)	0.98 (0.57–1.70)	0.952		
Occupation*						
Sex work	39 (13)	15 (38)	1.00 (0.50–2.01)	0.986		
Subsistence fisheries	64 (21)	20 (31)	0.67 (0.37–1.22)	0.189		
Salon/lodge/bar worker, market/street vendor	82 (27)	33 (40)	1.12 (0.66–1.88)	0.676		
Other occupation (professional, student, crafts, etc.)	130 (43)	52 (40)	1.13 (0.71–1.81)	0.604		
PrEP uptake						
No	219 (73)	87 (40)	Ref			
Yes	81 (27)	28 (35)	0.80 (0.47–1.36)	0.415		
Calendar period at 1-year assessment						
Pre-COVID (up to March 2020)	206 (69)	75 (36)	Ref			
Post COVID (beyond March 2020)	94 (31)	40 (43)	1.29 (0.79–2.13)	0.310		

*Multiple options allowed; ^#^An increase in risk score implied higher risk at the 1-year assessment.

## Discussion

Overall, in this cohort of participants deemed to be at high risk of HIV acquisition, there was a reduction in self-reported risky sexual behaviour indicators over a 1-year period. This study accorded participants, helped by a counsellor, an opportunity to identify high-risk behaviours that they engaged in. Through person-centred counselling, study counsellors supported and guided participants to realise how such behaviours put them at increased risk of acquiring HIV and discussed the methods of HIV prevention that the participant could take up to reduce the risk. Previous recommendations from the American Centre for Disease Control suggested that counselling in healthcare settings may not have an effect on HIV risk [16]. Further, evidence from a systematic review by Zajac and colleagues demonstrated that the impact of behavioural counselling on sexual risk behaviour in low- and middle-income countries remains inconclusive [7]. It is possible, however, that as time passes and HIV awareness increases even as the number of effective prevention interventions increases, the impact of behavioural counselling may become more vivid.

The biggest difference was observed in the proportion of participants who reported unprotected sex with three or more sexual partners, followed by that for those who reported having been diagnosed or treated for STIs in the last 3 months. Reductions were also observed in the proportion of participants who reported transactional sex, had six or more partners, and those who had sex while drunk. There is evidence from elsewhere that behavioural counselling may play a role in reducing risky behaviour [17]. Bolu et al. demonstrated a reduction in sexually transmitted disease diagnoses among high-risk subjects after the implementation of an HIV/STD counselling strategy [18]. In our study, provision of testing and treatment services for STIs could also have contributed to subsequent reduction in diagnosis and treatment for the same. We did not observe significant improvement in the prevalence of condom use at last sex and use of recreational drugs between baseline and 1 year. Condom use is well known to reduce the risk of HIV acquisition, while recreational drugs have also been determined to increase the risk of HIV [19,20]. Condom use is hampered by a number of factors including accessibility [21] as well as partner approval, especially among women who are in male-dominated relationships or who are financially dependent on their partner [22,23]. The factors that perpetuate recreational drug use in Africa need further exploration but have been suggested to include the predominance of youth in the demographic profile as well as growing urbanisation [24]. Programmes like our own that offer behavioural change counselling may need to give more attention to these behaviours to increase chances of reducing HIV risk.

While this risk reduction counselling may not be easily implementable in a routine health facility due to constraints such as extensive time requirements, clinics or sections of clinics particularly established to handle high-risk individuals may consider offering a similar package. Making it regular may render it more likely to be beneficial.

It is remarkable to note that all risk sexual behaviours were more prevalent at baseline among those that subsequently took up PrEP. This was probably because these individuals perceived their HIV risk to be high and were, therefore, willing to take up measures to reduce this risk including PrEP. This shows that measures to initiate PrEP may be more successful if they are targeted to individuals at highest risk of HIV acquisition. It has been demonstrated elsewhere that individuals that perceive themselves to be at risk are more likely to take up PrEP [25]. Another paper previously published from our cohort showed that individuals with ≥6 sex partners and those engaging in transactional sex were most likely to initiate PrEP [26].

Interestingly, we found that PrEP uptake was not associated with unchanged/increased risk behaviour. It was previously feared that a decrease in perceived risk due to the use of effective HIV prevention measures such as PrEP and safe male circumcision could lead to engagement in high-risk behaviour and consequently remove the benefit of the intervention [27,28]. Our finding is consistent with those of several other studies that have demonstrated that PrEP does not cause risk compensation [29–33]. This message should be integrated in the packages for the promotion of PrEP uptake.

It is worth noting that this study enrolled more males than females. It is quite common in this setting for studies to enrol more males than females due to a number of reasons, among which is the resentment of females to use reliable contraception during study participation, which is a common requirement in vaccine/therapeutic trials and cohorts set up to prepare for such trials [34,35]. It has also been postulated that females may require permission from their partners prior to participation in research, unlike males [36].

We would also like to clarify that while quite many participants were enrolled in the cohort, only 300 were included in this analysis. A number of reasons were responsible for this, such as loss of desire by participants to continue in the cohort, change of location, and loss to follow-up [37], among others. The COVID-19 situation in the same period most likely also impacted follow-up. Also important, when the clinical trial started enrolling in December 2020, participants could be moved from the cohort before completing 1 year as long as they met trial eligibility criteria.

## Limitations

A major limitation of this study is that the data were generated from self-reports. For example, reported diagnosis or treatment for STIs was not confirmed by review of medical records. This kind of information is often affected by participation and response biases, such as intentional misrepresentation (social desirability bias) and inaccurate recall [38]. The impact of any recall bias is, however, likely to have been minimised by the short recall period (≤3 months). Social desirability bias was reduced by using an experienced research team that is familiar with working in this kind of population, and we ensured confidentiality among participants. The risk score used in this analysis is not conventional and/or has not been used elsewhere. We, however, used factors that are known risk factors for HIV and so the score should be able to give good quantifiable estimate of risk. Social desirability bias is a potential occurrence in this kind of study. Lastly, some participants were not included in this analysis as indicated in Figure 1, because they missed the 1-year assessment, had not completed 1 year yet, or were terminated before 1 year. We are not sure how the results would have turned out if they had been included in this analysis. Despite these limitations, our study demonstrated the feasibility of reducing HIV risk behaviours among key populations through regular person-centred counselling delivered by lay counsellors.

## Conclusion

We observed a reduction in self-reported high-risk sexual behaviour indicators in this cohort of participants who were offered a regular person-centred risk reduction counselling. There was no evidence of risk compensation among participants who initiated PrEP. Regular individualised risk-reduction counselling should be considered for individuals at high risk of HIV acquisition in addition to PrEP and other HIV prevention interventions.

## Supplementary Material

Supplemental Material

## Data Availability

Data supporting the conclusions of this article can be retrieved through the link: https://doi.org/10.17037/DATA.00003403.

## Paper context

Evidence on the impact of counselling on risky sexual behaviour remains inconclusive. There are also concerns that the use of PrEP for HIV prevention may cause risk compensation. We observed a reduction in high-risk sexual behaviour indicators among participants who received regular person-centred risk reduction counselling, without evidence of risk compensation among participants who initiated PrEP. Regular individualised risk-reduction counselling should be considered for individuals at high risk of HIV acquisition in addition to PrEP.

## Members of the PrEPVacc study group

Glenda Gray Nishanta Singh, Zakir Gaffoor, Neetha Morar, Thandiwe Sithole, Kubashni Woeber, Samantha Siva, Eldinah Hwengwere, Sinqobile Nzama, Mlungisi Zwane, Hlobisile Gumede, Nompumelelo Ngcobo, Thasha Gounden, Vumile Gumede, Nokwazi Ntuli, Elizabeth Spooner, Ncengani Mthethwa, Sibusiso Nhleko, Veronica Chamane, Olive Gumede, Rabia Imamdin, Kerusha Chunderduri, Tamon Cafun-Naidoo, Victoria Mathenjwa, Clifford Gcwensa, Ilesh Jani, Edna Viegas, Isabel Remane, Odete Bule, Edna Nhacule, Patricia Ramgi, Raquel Chissumba, Eduardo Namalango, Yolanda Manganhe, Carmelia Massingue, Igor Capitine, Jorge Ribeiro, Lucas Maganga, Wiston William, Emmanuel Kapesa, Elizabeth Danstan, Doreen Pamba, Marco Missanga Amani Kway, Abisai Kisinda, Lilian Njovu, Lwitiho Sudi, Revocatus Kunambi, Jane Ambindwile, Nhamo Chiwerengo, Said Aboud, Patricia Munseri, Eligius Lyamuya, Frank Msafiri, Agricola Joachim, Edith Tarimo, Diana Faini Tumaini Nagu, Deus Buma, Muhammad Bakari, Moses Lufundya, Moshi Bilingo, Colman Mchau, Helen Mtui, Rujeko Samanthia Chidawanyika, Nteboheleng Mahapa, Phindile Khanyile, Londiwe Shandu, Silindile Zulu, Pontiano Kaleebu, Berna Kalanzi, Freddie Mukasa Kibengo, Ayoub Kakande, Jennifer Serwanga, Janet Seeley, Rachael Kawuma, Christian Holm Hansen, Sheila Kansiime, Eugene Ruzagira, Sylvia Kusemererwa, Sylvia Masawi, Vincent Basajja, Tobias Vudriko, Peter Hughes, Shamim Nabukenya, Gertrude Mutonyi, Rita Nakiboneka, Susan Mugaba, Ubaldo Bahemuka, Ben Gombe, Jane Nabbuto, Phiona Kaweesi, Wilson Kakeeto, Dorothy Abigaba, Bridget Muhofa, Kyeyune Mugagga, Jonathan Kitonsa, Lhwanzu Kitooke, Sylvia Masawi, Jonathan Weber, Cherry Kingsley, Tom Miller, Julie Fox, Sheena McCormack, Angela Crook, David Dunn, Henry Bern, Aminata Sy, Liz Brodnicki, Anna Griffiths, Simona Salomone, Sarah Joseph, Claire Wenden, Arianna Marini, Jonathan Hare, Claire Streafield, Kundai Chinyenze, Ansuya Naidoo, Jacqueline Musau, Mabela Matsoso, Mary Amondi, Paramesh Chetty, Anne Gumbe, Brian Kabuubi, Tabitha Wambugu, Giuseppe Pantaleo, Song Ding, Charlotta Nilsson, Arne Kroidl, Chriss Geldmacher, Kathrin Held, Gustavo Doncel, Allison Matthews, Jim Rooney, Alex Kintu, Carter Lee, Vineeta Gulati, Merlin Robb.
