# Supplementary material for: Changes in self-reported risky sexual behaviour indicators among adults receiving regular risk reduction counselling and optional initiation of pre-exposure prophylaxis in an HIV vaccine preparedness study in Masaka, Uganda
Source: Glob Health Action. 2023 Aug 7;16(1):2242672. doi: 10.1080/16549716.2023.2242672 (PMC10408567; doi:10.1080/16549716.2023.2242672)
Supplement: Supplemental Material [file ZGHA_A_2242672_SM7210.zip › S1_Risk_assesment_questionnaire_bot_6.pdf]

Study ID: Protocol code: P 0 1 | Site code: ☐☐ | Participant ID: ☐☐☐☐

Visit date: ☐☐ (dd) ☐☐☐ (MMM) ☐☐☐☐ (yyyy)

Visit number: ☐☐ . ☐

Complete at enrolment (month 0) and months 6, 12, 18, 24, 30, and 36

No	Code	Questions and filters	Coding categories	Skip
1.	LSEX	Wesemba ddi okutabagana mu bikolwa eby'ekyaama?<br>***When did you last have sex?***	≤1 week ☐<br>>1 to ≤4 weeks ☐<br>>4 weeks to ≤3 months ☐<br>>3 months ☐	**If >3 months, skip Q4-18, 20, 21, 31, & 34**
2.	LSEXC	Omulundi gwe wasembayo okutabagana mu bikolwa eby'ekyama, wakozesa kondomu?<br>***Did you use a condom the last time you had sex?***	No ☐<br>Yes ☐<br>Not sure ☐	**If No or Not sure, skip to Q4**
3.	LSEXNC	Oba yee, ddi lwe wasemba okutabagana mu bikolwa eby'ekyaama nga tokozeseza kondomu?<br>***If yes, when was the last time you had sex without a condom?***	≤1 week ☐<br>>1 to ≤4 weeks ☐<br>>4 weeks to ≤3 months ☐<br>>3 months ☐	
4.	NPART	Mu myezi esatu egiyise, otabaganye n'abantu bameka mu bikolwa eby'ekyaama?<br>***How many persons did you have sex with in the last 3 months? (please encourage respondent to give an approximate number if he/she cannot remember exact number)***	☐☐☐	
5.	PCON	Mu myezi esatu egiyise, bantu bameka b'otabaganye nabo mu bikolwa eby'ekyaama nga tokozesezza kondomu?<br>***How many persons did you have sex with without using a condom in the last 3 months?***	☐☐	
6.	NEWP	Ku bantu be watabaganye nabo mu bikolwa eby'ekyaama mu myezi esatu egiyise, bameka abaali abapya (be wali totabaganangako nabo)?<br>***How many of the persons you had sex with in the last 3 months were new sexual partners i.e. persons you had never had sex with before?***	☐☐	**If '00', skip to Q8**
7.	CNEWP	Ku bantu abapya b'otabaganye nabo mu myezi esatu egiyise, bameka ku bo bewatabagana nabo nga tokozesezza kondomu?<br>***How many of your new sexual partners in the last 3 months did you have sex with without using a condom?***	☐☐	
8.	TRA	Mu myezi esatu egiyise, owereddwa oba olina gw'owadde ssente, ebirabo oba embeera endala yonna nga kigendereddwamu okwegatta mu bikolwa eby'ekyaama n'omuntu oyo?<br>***In the last 3 months, have you received or given money, gifts, or other favours in exchange for having sex with someone?***	No ☐<br>Yes, received ☐<br>Yes, given ☐<br>Yes, both ☐	**If No, skip to Q11**
9.	TRAP	Oba yee, ddi lwe wasemba okufuna oba okuwa ssente, ebirabo oba okubweebwa obuyambi obw'engeri yonna nga bino bigendereddwamu okwegatta mu bikolwa eby'ekyaama n'omuntu oyo?<br>***If yes, when was the last time that you received/gave money, gifts, or other favours in exchange for having sex with someone?***	≤1 week ☐<br>>1 to ≤4 weeks ☐<br>>4 weeks to ≤3 months ☐	

Study ID: Protocol code: P 0 1 | Site code: ☐☐ | Participant ID: ☐☐☐☐

Visit date: ☐☐ (dd) ☐☐☐ (MMM) ☐☐☐☐ (yyyy)

Visit number: ☐☐ . ☐

No	Code	Questions and filters	Coding categories	Skip
10.	TRAC	Wakozesa kondomu lwe watabagana mu bikolwa eby'ekyaama n'omuntu gwe wawa oba, eyakuwa ssente, ebirabo oba okuweebwa obuyambi obwengeri yonna endala nga bino bigendereddwamu okukusendasenda okwegatta naye mu bikolwa eby'ekyaama?<br>***Did you use a condom when you had sex in exchange for money, gifts, or other favours?***	Never used a condom ☐<br>Yes, always ☐<br>Yes, sometimes ☐	
11.	SXUK	Mu myezi esatu egiyise, olina omuntu gwe wetabye naye mu bikolwa eby'ekyaama nga tomanyi bw'ayimiridde ku bya kawuka ka siliimu?<br>***In the last 3 months, have you had sex with persons whose HIV status you do not know?***	No ☐<br>Yes ☐	**If No, skip to Q14**
12.	NSXUK	Oba yee, bantu bameka b'otamanyi bwe bayimiridde ku bya kawuka ka siliimu be watabagana nabo mu bikolwa eby'ekyaama mu myezi esatu egiyise?<br>***If yes, how many persons whose HIV status you do not know have you had sex with in the last 3 months?***	☐☐	
13.	SXUKC	Ng'otabagana n'abantu b'otomanyi mbeera zaabwe ku kawuka ka siliimu, wakozesa kondomu ?<br>***Did you use a condom when you had sex with this/these person(s) whose HIV status you do not know?***	Never used a condom ☐<br>Yes, always ☐<br>Yes, sometimes ☐	
14.	SXPOS	Mu myezi esatu egiyise, watabaganako n'omuntu alina akawuka ka siliimu?<br>***In the last 3 months, have you had sex with someone who is HIV-infected?***	No ☐<br>Yes ☐<br>Don't Know ☐	**If No / Don't know, skip to Q16**
15.	SXPOSC	Obe yee, wakozesa kondomu ng'otabagana n'omuntu ono alina akawuka ka siliimu?<br>***If yes, did you use a condom when you had sex with this HIV-infected person?***	Never used a condom ☐<br>Yes, always ☐<br>Yes, sometimes ☐	
16.	SXOLD	Mu myezi esatu egiyise, otabaganyeko n'omuntu akusiinga obukulu emyaka 10 oba n'okusingawo?<br>***In the last 3 months, have you had sex with someone older than you by 10 years or more?***	No ☐<br>Yes ☐<br>Not sure ☐	
17.	OPART	Olina omuntu gw'otabaganye naye mu bikolwa eby'ekyaama mu myezi esatu egiyise ate nga okimanyi nti alina abantu abalala batabagana nabo?<br>***Do you know if any of your sexual partners in the last 3 months has other partners?***	No ☐<br>Yes ☐<br>Not sure ☐<br>Don't Know ☐	

Study ID: Protocol code: P 0 1 | Site code: [ ][ ] | Participant ID: [ ][ ][ ][ ]

Visit date: [ ][ ] dd [ ][ ][ ] MMM [ ][ ][ ][ ] yyyy

Visit number: [ ][ ] . [ ]

No	Code	Questions and filters	Coding categories	Skip
18.		Mu myezi esatu egiyise, olina lwe wetabagana mu bikolwa eby'ekyaama nga;<br>***In the last 3 months, have you had sex when;***	No Yes	
	UWSEX	• Nga toyagala naye ne kibaawo?<br>***You did not want to have sex, but it happened?***	☐ ☐	
	PSEX	• Nga toyagala kwetabamu naye n'okikola olw'okuwalirizibwa?<br>***You did not want to have sex, but felt pressurised?***	☐ ☐	
	FSEX	• Nga toyagala kwetabamu naye n'okakibwa okukikola?<br>***You did not want to have sex, but were physically forced?***	☐ ☐	
19.	ASM	**Abasajja bokka:** Wali wegasseeko mu bikolwa eby'ekyaama ne basajja bano *(okulya ebisiyaga)*?<br>***Men only: Have you ever anal sex with other men?***	No ☐<br>Yes ☐	**If No, skip to Q22**
20.	RASM	**Abasajja bokka:** Oba yee, wegaseeko mu bikolwa eby'ekyaama ne basajja bano *(okulya ebisiyaga)* mu myezi esatu egiyise?<br>***Men only: If yes, have you had anal sex with other men in the last 3 months?***	No ☐<br>Yes ☐	**If No, skip to Q22**
21.	CRASM	**Abasajja bokka:** Oba yee, wakozesa kondomu ng'otabagana mu bikolwa eby'ekyaama *(okulya ebisiyaga)* ne basajja bano?<br>***Men only: If yes, did you use a condom when you had anal sex with other men?***	Never used a condom ☐<br>Yes, always ☐<br>Yes, sometimes ☐	
22.	DISCH	Mu myezi esatu egiyise, wafunyeeko okuvaamu amazzi oba ekintu ekitali kya bulijjo mu bitundu byo eby'ekyaama?<br>***In the last 3 months, have you had any abnormal genital discharge?***	No ☐<br>Yes ☐<br>Not sure ☐	**If No /Not sure, skip to Q24**
23.	DISCHP	Oba yee, ddi lwe wasemba okuvaamu amazzi oba ekintu ekitali kya bulijjo mu bitundu byo eby'ekyaama?<br>***If yes, when was the last time you had an abnormal genital discharge?***	≤1 week ☐<br>>1 to ≤4 weeks ☐<br>>4 weeks to ≤3 months ☐	
24.	ULCER	Mu myezi esatu egiyise, wafunako okusalikasalika, ebiwundu oba mu bitundu byo eby'ekyaama?<br>***In the last 3 months, have you had genital sores or ulcers?***	No ☐<br>Yes ☐<br>Not sure ☐	**If No /Not sure, skip to Q26**
25.	ULCERP	Oba yee, ddi lwe wasembayo okuba n'ebiwundu oba amabwa mu bitundu byo?<br>***If yes, when was the last time you had genital sores or ulcers?***	≤1 week ☐<br>>1 to ≤4 weeks ☐<br>>4 weeks to ≤3 months ☐	

Study ID: Protocol code P 0 1 | Site code | Participant ID

Visit date: dd MMM yyyy

Visit number: .

No	Code	Questions and filters	Coding categories	Skip
26.	STIS	Mu myezi egiyise, baali bakukebeddeko n'osangibwa nga olinaendwadde y'ekyaama oba n'ofuna obujjanjabi bw'enddwadde y'ekyaama?<br>***In the last 3 months, have you been diagnosed with or received treatment for a sexually transmitted infection?***	No ☐<br>Yes ☐<br>Don't know/Not sure ☐	**If No /Don't know /Not sure , skip to Q28**
27.	STISP	Oba yee, ddi lwe wasembayo okukeberebwa n'osangiba n'endwadde y'ekyaama oba n'owebwa obujjanjabi bw'endwadde y'ekyaama?<br>***If yes, when was the last time you were diagnosed with or received treatment for a sexually transmitted infection?***	≤1 week ☐<br>>1 to ≤4 weeks ☐<br>>4 weeks to ≤3 months ☐	
28.	STIP	Mu myezi esatu egiyise, waliwo ku baagalwa bo gwe baakebera n'asangibwa n'endwadde oba naawebwa obujjanjabi bw'endwadde y'ekyaama?<br>***In the last 3 months, have any of your partners been diagnosed with or received treatment for a sexually transmitted infection?***	No ☐<br>Yes ☐<br>Don't know/Not sure ☐	**If No /Don't know /Not sure , skip to Q30**
29.	STIPP	Oba yee, ddi omwagalwa wo lwe yasembayo okusangibwa n'endwadde oba okuweebwa obujjanjabi bw'endwadde y'ekyaama?<br>***If yes, when was the last time your partner was diagnosed with or received treatment for a sexually transmitted infection?***	≤1 week ☐<br>>1 to ≤4 weeks ☐<br>>4 weeks to ≤3 months ☐	
30.	DRUG	Mu myezi esatu egiyise, wakozesaako ebiragalalagala olw'okunyumirwa oba olw'ekwesanyusaamu nga si lwa bujjanjabi?<br>***Have you used any recreational drugs (i.e. chemical substances taken for enjoyment, or leisure purposes, rather than for medical reasons) in the last 3 months? (Please give examples of the commonly used recreational drugs in the study area)***	No ☐<br>Yes ☐	**If No , skip to Q32**
31.	DRUSX	Oba yee, wetabye mu bikolwa by'okutabagana kyenkana ki oluvannyuma lw'okukozesa ebiragalalagala?<br>***If yes, how often did you have sex after using recreational drugs?***	Never ☐<br>Sometimes (<50% of the time) ☐<br>Frequently (>50% of the time) ☐<br>Always ☐	
32.	ALCH	Mu myezi esatu egiyise, mirundi emeka gy'onywedde eky'okunywa omuli ekitamiiza?<br>***In the last 3 months, how often did you have a drink containing alcohol?***	Never ☐<br>Once a month or less ☐<br>2-4 times a month ☐<br>2-3 times a week ☐<br>4 or more times a week ☐	**If Never, skip to Q35**
33.	DRNK	Ku mirundi gye wanywa eky'okunywa omuli ekitamiiza, bitundu byenkana wa ku mirundi egyo lwe watamiira?<br>***How often did you get drunk after having an alcoholic drink?***	Never ☐<br>Sometimes (<50% of the time) ☐<br>Frequently (>50% of the time) ☐<br>Always ☐	**If Never, skip to Q35**

Study ID: Protocol code: P 0 1 | Site code: ☐☐ | Participant ID: ☐☐☐☐

Visit date: ☐☐ (dd) ☐☐☐ (MMM) ☐☐☐☐ (yyyy)

Visit number: ☐☐ . ☐

No	Code	Questions and filters	Coding categories	Skip
34.	DRNKSX	Ku mirundi ki gy'otabaganye mu bikolwa eby'ekyaama, geraageranya ku gyo, kyenkana ki lwewakikola nga otamide?<br>***How often did you have sex when drunk?***	Never ☐<br>Sometimes (<50% of the time) ☐ ☐<br>Frequently (>50% of the time) ☐<br>Always ☐	
35.	CIRC	Oli mukomole?<br>***Are you circumcised?***<br>***(If female, indicate Not Applicable)***	No ☐<br>Yes ☐<br>Not Applicable ☐	
36.	KPREP	Wali owuliddeko enkola ya PrEP; enkola ya PrEP yeeyo abantu abatalina kawuka ka siliimu mwebakozeseza eddagala erikajjanjaba kibayambe okuziyiza okukwatibwa akawuka ka siliimu?<br>***Have you heard about pre-exposure prophylaxis (PrEP) i.e. the use of anti-HIV drugs by HIV-negative persons to protect themselves from catching HIV?***	No ☐<br>Yes ☐	**If No, skip to Q40**
37.	UPREP	Wetwogerera kati okozesa enkola eya PrEP?<br>***Are you currently using PrEP?***	No ☐<br>Yes ☐	**If No, skip to Q40**
38.	SPREP	PrEP omufuna kuva wa?<br>***Where do you get PrEP from?***	Public/government clinic ☐<br>NGO clinic ☐<br>Private clinic ☐<br>Other ☐	
39.	SSPREP	Nnyonnyola ebifo ebirala awasangibwa PrEP<br>***Specify other source of PrEP***		
40.	WUPREP	Wandikkirizza okukozesa enkola ya PrEP singa eba ekuweereddwa?<br>***Would you be willing to use PrEP if it were offered to you?***	No ☐<br>Yes ☐<br>Not sure ☐	
41.	CONTR	Olina enkola eziyiza okufuna olubuto gy'okozesa kati?<br>***Are you currently using any contraceptive method?***<br>***(If male, indicate Not Applicable and skip to Q44)***	No ☐<br>Yes ☐<br>Not Applicable ☐	**If No, skip to Q44**
42.		Nkola ki ey'okwetangira okufuna olubto gy'okozesa kati?<br>***What methods of contraception are you currently using? (multiple responses allowed)***	No / Yes	
	ORAL	Okumira empeke<br>***Oral contraceptives***	☐ / ☐	
	PATCH	Ekipaapi ky'oku mukono<br>***Transdermal patch***	☐ / ☐	
	INJDEP	Empiso eya buli myezi esatu<br>***Injectable-DMPA contraceptives***	☐ / ☐	
	INJNET	Empiso eya buli myezi<br>***Injectable-NET-EN***	☐ / ☐	
	INJCOMB	Empiso erimu eddagala ly'ebika ebibiri<br>***Injectable-combined progestin-oestrogen***	☐ / ☐	

Study ID: Protocol code P 0 1 | Site code ☐☐ | Participant ID ☐☐☐☐

Visit date: ☐☐ dd ☐☐☐ MMM ☐☐☐☐ yyyy

Visit number: ☐☐ . ☐

No	Code	Questions and filters	Coding categories	Skip
	IMPLANT	Obuti obuteekebwa mu mukono<br>***Implant***	☐ ☐	
	IUD	Akaweta k'omunnabaana<br>***Intrauterine device***	☐ ☐	
	SURG	Okukomezebwa nga basala obuseke<br>***Surgical sterilisation***	☐ ☐	
	MCOND	Obupiira bw'ekisajja<br>***Male condoms***	☐ ☐	
	FCOND	Obupiira bw'ekikyaala<br>***Female condoms***	☐ ☐	
	SPERM	Eddagala eritta enkwaso y'omusajja<br>***S***permicides	☐ ☐	
	OTHER	Enkola endala yonna<br>***Other***	☐ ☐	
43.	SOTHER	Bw'eba enkola endala yonna nnyonnyola<br>***If other contraceptive method, specify***		
44.	STAFR	Staff initials	☐☐☐	

<u>Not for data entry</u>

Reviewed by:

________________ Initials ________________ Date

1st data entry performed by:

________________ Initials ________________ Date

2nd data entry performed by:

________________ Initials ________________ Date
