# Supplementary material for: Changes in self-reported risky sexual behaviour indicators among adults receiving regular risk reduction counselling and optional initiation of pre-exposure prophylaxis in an HIV vaccine preparedness study in Masaka, Uganda
Source: Glob Health Action. 2023 Aug 7;16(1):2242672. doi: 10.1080/16549716.2023.2242672 (PMC10408567; doi:10.1080/16549716.2023.2242672)
Supplement: Supplemental Material [file ZGHA_A_2242672_SM7210.zip › S2_A_comparison_of_participants_wit_7.docx]

**S2: A comparison of participants with 1-year follow up and those without**

| **Socio-demographic characteristics** | | **Participants with follow up** | **Participants without follow up** | **All enrolled participants** | **P-value**** |
| --- | --- | --- | --- | --- | --- |
|  |  | N (%)† | N (%)† | N (%)† |  |
| All | | 300 (100) | 812 (100) | 1112 (100) |  |
| Gender | |  |  |  |  |
|  | Male | 168 (56) | 177 (22) | 345 (31) |  |
|  | Female | 132 (44) | 635 (78) | 767 (69) | P<0.001 |
| Age category | |  |  |  |  |
|  | ≤24 | 152 (51) | 479 (59) | 631 (57) |  |
|  | 25-34 | 114 (38) | 298 (37) | 412 (37) |  |
|  | ≥35 | 34 (11) | 35 (4) | 69 (6) | P<0.001 |
| Education | |  |  |  |  |
|  | ≤Primary | 195 (65) | 463 (57) | 658 (59) |  |
|  | ≥Secondary | 105 (35) | 349 (43) | 454 (41) | 0.016 |
| Marital status | |  |  |  |  |
|  | Single | 142 (48) | 436 (54) | 578 (52) |  |
|  | Married/cohabiting/in a relationship | 121 (40) | 176 (22) | 297 (27) |  |
|  | Divorced/separated/widowed | 37 (12) | 200 (25) | 237 (21) | P<0.001 |
| Religion | |  |  |  |  |
|  | Christian | 229 (76) | 603 (74) | 832 (75) |  |
|  | Muslim/other | 71 (24) | 209 (26) | 280 (25) | 0.475 |
| Occupation* | |  |  |  |  |
|  | Sex work | 39 (13) | 414 (51) | 453 (41) | P<0.001 |
|  | Subsistence fisheries | 64 (21) | 38 (5) | 102 (9) | P<0.001 |
|  | Salon/lodge/bar worker, market/street vendor | 82 (27) | 261 (32) | 343 (31) | 0.124 |
|  | Other occupation (professional, student, crafts etc.) | 130 (43) | 217 (27) | 347 (31) | P<0.0001 |
| Electricity in household | |  |  |  |  |
|  | No | 82 (27) | 265 (33) | 347 (31) |  |
|  | Yes | 218 (73) | 547 (67) | 765 (69) | 0.093 |

^†^Column percentage; *Multiple options allowed; **Chi square P-value
