# Supplementary material for: Changes in self-reported risky sexual behaviour indicators among adults receiving regular risk reduction counselling and optional initiation of pre-exposure prophylaxis in an HIV vaccine preparedness study in Masaka, Uganda
Source: Glob Health Action. 2023 Aug 7;16(1):2242672. doi: 10.1080/16549716.2023.2242672 (PMC10408567; doi:10.1080/16549716.2023.2242672)
Supplement: Supplemental Material [file ZGHA_A_2242672_SM7210.zip › S3_Risk_Scores.docx]

**S3: Risk scores (at baseline, 6 months and 1 year)**

| **Risk score** | **Baseline**  **Participants (%)** | **6 months Participants (%)**** | **1 year**  **Participants (%)** |
| --- | --- | --- | --- |
| 0 | 3 (1) | 24 (8) | 18 (6) |
| 1 | 31 (10) | 81 (29) | 88 (29) |
| 2 | 64 (21) | 80 (28) | 81 (27) |
| 3 | 74 (25) | 53 (19) | 54 (18) |
| 4 | 58 (19) | 28 (10) | 29 (10) |
| 5 | 51 (17) | 12 (4) | 20 (7) |
| 6 | 16 (5) | 3 (1) | 9 (3) |
| 7 | 3 (1) | 2 (1) | 1 (0) |
| Overall | 300 (100) | 283 (100) | 300 (100) |

*Overall, there was a decrease in risk scores at 1 year in comparison to baseline.

**Of the 300 participants assessed at 1 year, 17 participants missed their 6 months assessment.
